# Do we really need to differentiate mesenchymal stem cells into insulin-producing cells for attenuation of the autoimmune responses in type 1 diabetes: immunoprophylactic effects of precursors to insulin-producing cells

**DOI:** 10.1186/s13287-017-0615-1

**Published:** 2017-07-12

**Authors:** Anshu Sharma, Rajni Rani

**Affiliations:** 1 Molecular Immunogenetics Group, National Institute of Immunology, New Delhi, 110067 India; 2 Systems Biology Group, CSIR—Institute of Genomics and Integrative Biology, New Delhi, 110025 India

**Keywords:** Mesenchymal stem cells (MSCs), Insulin-producing cells (IPCs), precursors to insulin-producing cells (pIPCs), Nonobese diabetic (NOD) mice

## Abstract

**Background:**

Type 1 diabetes (T1D) is a multifactorial autoimmune disorder where pancreatic beta cells are lost before the clinical manifestations of the disease. Administration of mesenchymal stem cells (MSCs) or MSCs differentiated into insulin-producing cells (IPCs) have yielded limited success when used therapeutically. We have evaluated the immunoprophylactic potentials of precursors to insulin-producing cells (pIPCs) and IPCs in nonobese diabetic (NOD) mice to ask a basic question: do we need to differentiate MSCs into IPCs or will pIPCs suffice to attenuate autoimmune responses in T1D?

**Methods:**

Bone marrow-derived MSCs from Balb/c mice were characterized following the International Society for Cellular Therapy (ISCT) guidelines. MSCs cultured in high-glucose media for 11 to 13 passages were characterized for the expression of pancreatic lineage genes using real-time polymerase chain reaction. Expression of the PDX1 gene in pIPCs was assessed using Western blot and fluorescence-activated cell sorting (FACS). Triple-positive MSCs were differentiated into IPCs using a three-step protocol after sorting them for cell surface markers, i.e. CD29, CD44, and SCA-1. Nonobese diabetic mice were administered pIPCs, IPCs, or phosphate-buffered saline (PBS) into the tail vein at weeks 9 or 10 and followed-up for 29–30 weeks for fasting blood glucose levels. Two consecutive blood sugar levels of more than 250 mg/dl were considered diabetic.

**Results:**

MSCs grown in high-glucose media for 11 to 13 passages expressed genes of the pancreatic lineage such as *PDX1*, *beta2*, *neurogenin*, *PAX4*, *Insulin*, and *glucagon*. Furthermore, Western blot and FACS analysis for PDX-1, a transcription factor necessary for beta cell maturation, confirmed that these cells were precursors of insulin-producing cells (pIPCs). NOD mice administered with pIPCs were better protected from developing diabetes with a protective efficacy of 78.4% (*p* < 0.009); however, administration of IPCs gave protective efficacy of 55% at the end of 28–30 weeks.

**Conclusions:**

Precursors to insulin-producing cells seem to have better potential to arrest autoimmune response in type 1 diabetes when administered before the onset of the disease in NOD mice. When translated to humans, autologous mesenchymal stem cells grown in high-glucose media for 10 to 13 passages may have beneficial effects in individuals at high risk of developing type 1 diabetes.

**Electronic supplementary material:**

The online version of this article (doi:10.1186/s13287-017-0615-1) contains supplementary material, which is available to authorized users.

## Background

Type 1 diabetes (T1D) is a very complex, multifactorial disorder characterized by selective autoimmune destruction of insulin-producing beta cells of the pancreas [1]. T1D accounts for about 5% of all diabetic cases reported. The most devastating part of the disease is that it is a quiet killer of the pancreatic beta cells where the clinical manifestations occur only when more than 90% of the beta cells are lost. Under these circumstances, the only choice of treatment left for the physician is daily insulin injections which takes care of the glucose metabolism but does not really treat the patient, as they are still dependent on insulin. However, it is not easy to determine the right dose of insulin; it may lead to hyperglycemia if not enough insulin has been administered. On the other hand, a dose higher than the physiological need of the patient may lead to hypoglycemia. Unmanaged or mismanaged diabetes can lead to various complications such as neuropathy, nephropathy, retinopathy, cardiac complications, keto-acidosis, and diabetic coma [2].

Several approaches have been adopted to treat T1D, with limited success. While transplantation of islet cells from fresh cadaveric donors has shown success, heavy immunosuppression is required due to the allogeneic nature of the graft which in turn may make them prone to severe opportunistic infections. Also, usually more than one donor is required. Hence, due to scarcity of pancreatic donors, it may not be an ideal choice of treatment [3, 4], warranting the need for alternate treatment strategies such as stem cell therapy.

Stem cell therapy seems to be the best option to take care of the insulin requirements of the patients. Different strategies have been evaluated to differentiate embryonic stem cells (ESCs) or mesenchymal stem cells (MSCs) into islet-like cells. While ESCs may be a good option, their use has been limited because of ethical issues associated with ESCs and also their potential to form tumorigenic cells [5]. MSCs, on the other hand, are a good alternative as ethical issues are not associated with them and autologous MSCs can be differentiated and transplanted back into the patients.

MSCs are adult stem cells known for their ability to differentiate into mesodermal lineages such as adipocytes and osteocytes, etc. [6], and ectodermal [7, 8] as well as endodermal lineages [9–11]. MSCs are also known for their immunomodulatory properties and have been used for the treatment of autoimmune diseases [12, 13]. Due to their immunomodulatory and multilineage differentiation potential, MSCs are good options for cell-based therapy for T1D [14, 15]. MSCs grown in high-glucose media become programmed to express genes related to pancreatic development [16] and may become precursors to beta-like cells. Further treatment with chemicals can help them differentiate into mature insulin-producing cells (IPCs). However, there are several challenging questions that remain unanswered and need to be addressed. For example, what is the lifespan of the differentiated beta cells in vivo after transplantation? Since the proliferative capacity of differentiated cells is reduced, will it be sufficient to transplant them once, or will repeated transplants be required? We hypothesized that transplantation of precursors of beta cells or immunomodulatory MSCs may be a better option than transplanting differentiated IPCs to arrest T1D. To check whether this hypothesis holds true in an in-vivo scenario, we have evaluated the outcome of intravenous administration of precursors to insulin-producing cells (pIPCs) and differentiated IPCs in female nonobese diabetic (NOD) mice and followed them for up to 29–30 weeks. NOD mice spontaneously develop human T1D-like symptoms with a higher incidence in female mice compared to males. Diagnostic symptoms, such as hyperglycemia and loss of body weight, may become evident in female NOD mice by the age of 12 weeks and, by the age of 20 weeks, about 60–80% of female mice become diabetic while only 20–30% of male NOD mice become diabetic by that age [17].

## Methods

### Isolation and expansion of MSCs in culture

All animal studies were conducted following clearance from the Institutional Animal Ethics Committee of the National Institute of Immunology, New Delhi, India, and experiments were performed in accordance with relevant guidelines and regulations. Balb/c mice aged 4–8 weeks were sacrificed via cervical dislocation, and bone marrows were isolated from the femur and tibia bones. The ends of the bones were cut and bone marrow cells were flushed out using high-glucose (25 mM) Iscove's modified Dulbecco's medium (IMDM; Sigma) with 5% fetal bovine serum (FBS; GIBCO), with the help of a 26.5 G needle. A single-cell suspension of the bone marrow cells was centrifuged at 300 g for 5 min and cell pellet thus obtained was resuspended in IMDM containing 15% FBS and cultured at an initial seeding density of 10 × 10^6^ cells/25 cm^2^ at 37 °C in humidified 5% CO_2_. After 3 days of culture, nonadherent cells were removed and partial media changes were performed when the media became acidic. Once the cells were 70–80% confluent, they were split into two flasks after trypsinization using 0.25% trypsin (in 1 mM EDTA) at 37 °C for 1 min. Trypsinization was stopped with serum containing medium, and cells were transferred to 15-ml conical centrifuge tubes and centrifuged at 300 g for 5 min. The cell pellet was resuspended in IMDM with 15% FBS and recultured further in two tissue-grade flasks to expand the number of cells.

### Phenotypic surface marker expression on MSCs using FACS

MSCs were harvested after 4–5 passages using trypsin-EDTA as described previously, washed in flow cytometry buffer (phosphate-buffered saline (PBS), 0.1% bovine serum albumin (BSA), 0.05% sodium azide) and were stained with the following antibodies for 30 min at 4 °C: fluorescein isothiocyanate (FITC)-conjugated hamster anti-rat CD29, phycoerythrin (PE) rat anti-mouse CD73, BV421-rat anti-mouse Ly-6A/E, PE-cy5 rat anti-mouse CD44 (BD Biosciences), biotin conjugated anti-mouse CD11b, CD34, and CD45 (eBiosciences). After staining the cells were washed and resuspended in flow cytometry buffer for further acquisition. Those cells which had been treated with biotinylated antibodies were further stained with PE-conjugated streptavidin for 30 min at 4 °C, washed, and resuspended in flow cytometry buffer. Flow cytometry was performed on a BD FACS Verse flow cytometer and analysis was performed with BD Flowjo software (version 5.0).

### Multilineage differentiation potential of MSCs

MSCs have the potential to differentiate into multiple lineages, and so to further characterize them we differentiated them into adipogenic and osteogenic lineages using standard protocols [18–20].

For adipogenic differentiation the cells were seeded at an initial seeding density of 3000 cells/cm^2^. Once the cells were 70–80% confluent they were treated with freshly prepared 0.521 mM isobutyle-1-methylxanthine (IBMX; made in 0.5 M KOH), 1 μM dexamethasone, and 5 μg/ml insulin in IMDM (15% FBS) for 3 days. After 3 days, the medium was changed to IMDM (15% FBS) containing 5 μg/ml insulin only and the cells were cultured in this insulin-containing medium for 11 days with a medium change every third day. After the completion of the induction protocol, the cells were stained with Oil red O dye which stains the neutral triglycerides and lipids as bright red in the multilocular oil droplets.

For osteogenic differentiation cells were cultured in IMDM with 50 μM ascorbate phosphate, 10 mM β-glycerol phosphate, and 10 nM dexamethasone for 28 days with medium changes every 3 days. After the completion of the induction protocol, cells were stained with Alizarin red dye stain which detects the intracellular calcium, calcium-binding proteins, and proteoglycans and is therefore ideal for demonstrating the mineralization in osteogenic differentiation [21, 22].

### Differentiation of MSCs into IPCs

#### Sorting the triple-positive (CD29^+^CD44^+^SCA-1^+^) MSC population

MSCs grown in high-glucose medium for 10–13 passages were trypsinized and resuspended in plain IMDM and sorted using fluorescence-activated cell sorting (FACS) to enrich the triple-positive cells having CD29, CD44, and SCA-1 phenotypic markers. To achieve this, the MSCs were stained with FITC-conjugated hamster anti-rat CD29 (FITC-CD29) (1:800), PE-cy5 rat anti-mouse CD44 (1:400), and brilliant violet 421 (BV421)-rat anti-mouse Ly-6A/E (SCA-1) (1:800) for 30 min at 4 °C in dark. After staining, cells were passed through a 70-μm cell strainer and centrifuged at 300 g for 5 min. In parallel, cells were also separately labeled with CD29, CD44, and SCA-1 surface markers for making the single color stain required for compensation at the time of cell sorting. Once the cells were ready for sorting they were resuspended in plain media and, thereafter, were sorted for triple-positive cell population using the FACS Aria cell sorter.

#### Differentiation of triple-positive MSCs into IPCs

MSCs triple positive for CD29, CD44, and SCA-1 were seeded in tissue culture flasks at a seeding density of 3000 cells/cm^2^ in IMDM containing 15% FBS and differentiated into IPCs using stage-specific differentiation as described by Chandra et al. [23] with a few modifications. After the cells were 60–70% confluent, the serum-containing medium was replaced with serum-free medium (SFM) containing 1% BSA (Sigma), and 1× insulin-transferrin-selenium (ITS) in IMDM in the control cells. However, cells for differentiation were cultured in induction medium SFM-A containing 4 nM activin A, 1 mM sodium butyrate, and 50 mM 2-mercaptoethanol in SFM and were cultured for 2 days in SFM-A. After 2 days of culture in SFM-A, medium was changed to SFM-B, containing 1% BSA, ITS, and 0.3 mM taurine in IMDM. Cells were cultured in this medium for 2 more days and medium was changed from SFM-B to SFM-C which contained 1.5% BSA, ITS, 3 mM taurine, 100 nM glucagon-like peptide (GLP)-1 (amide fragment 7–36; Sigma Aldrich), 1 mM nicotinamide, and 1× nonessential amino acids (NEAAs) in IMDM. SFM-C medium was changed after every second day of culture. GLP-1 was reconstituted after every 24 h due to its proteolytic degradation in the culture medium. It should be noted that in both control and induced media ITS was present. All chemicals were purchased from Sigma Aldrich.

### Culturing of pIPCs

The MSCs grown in IMDM containing 25 mM glucose for 10–13 passages were considered to be precursors to insulin-producing (pIPCs) cells and were characterized the same.

### Immunostaining of IPCs

After the completion of the induction protocol for insulin production, the IPCs and the cells grown in high-glucose medium (pIPCs) were stained for the presence of intracellular insulin. Cells were washed using PBS and fixed in 4% paraformaldehyde for 10 min at room temperature. After fixation, cells were washed with PBS followed by permeabilization using 0.25% Triton X-100 in PBS for 10 min and blocked using 1% BSA in PBS for 1 h at room temperature. For studying the presence of intracellular insulin, cells were incubated overnight with anti-insulin rabbit polyclonal antibody at 1:200 dilution (Santa Cruz SC-9168), washed with PBS, and stained with secondary antibody Alexa Fluor 488 goat anti-rabbit (Invitrogen) for 1 h at room temperature at 1:500 dilution. After another PBS wash, cells were counterstained for nuclei with DAPI (molecular probes) and observed under a fluorescent microscope.

### Western blot for PDX-1 protein

A Western blot was performed to quantify the expression of PDX-1 protein in MSCs grown in high-glucose media. Briefly, whole cell protein was extracted by the addition of RIPA lysis buffer. Protein samples were run on SDS polyacrylamide (10%) gel electrophoresis and transferred to PVDF membranes. Immunoblot was blocked with 5% nonfat dry milk (NFDM) for 1 h at room temperature and probed overnight with rabbit polyclonal PDX-1 antibody (1:10,000) (ab98298, Abcam) at 4 °C. The blot was then probed with horseradish peroxidase-conjugated anti-rabbit secondary antibody for 1 h at room temperature. ECL reagent (GE Healthcare) was added and the signal was detected on an X-ray photographic film which was developed and fixed in the dark. After stripping, the membrane was stained for HSP90 as a loading control.

### Intracellular FACS staining for the presence of PDX1

MSCs from the cultures grown in high-glucose media were obtained after trypsinization and were fixed in 4% paraformaldehyde, permeabilized using 0.25% Triton X-100 in PBS for 10 min, and blocked using 1% BSA in PBS for 1 h at room temperature. After blocking, cells were stained with anti-PDX1 antibody for 1 h at room temperature, washed with PBS. and stained with secondary antibody Alexa Fluor 488 goat anti-rabbit for 1 h at room temperature. Cells were resuspended in flow cytometry buffer and further run on a BD FACS Verse flow cytometer. Analysis was performed using BD Flowjo software (version 5.0).

### Transcriptional analysis using real-time polymerase chain reaction

RNA was isolated from the MSCs using Tri-reagent following the manufacturer’s protocol. The RNA was column purified using the Qiagen RNeasy Mini Kit and quantified using Nanodrop and run on a 1% agarose gel in TBE to determine RNA integrity (see Additional file 1 for a detailed protocol).

Furthermore, RNA was reverse transcribed using the Superscript III cDNA synthesis kit (Invitrogen) according to the manufacturer’s protocol (see Additional file 1). Gene expression analysis by real-time polymerase chain reaction (PCR) was carried out on the Applied Biosystems 7500 fast system. Briefly, 20 μl reactions in triplicate with 5–100 ng RNA equivalent of *cDNA* per reaction (in 5 μl) were set up using 10 μl ABI SYBR green master mix (2×) and 700 nM of forward and reverse primers. Primers for target genes were designed using the NCBI primer-blast checked for secondary structure formation and/or primer dimer formation using Gene Runner software. All the gene expressions were normalized to endogenous control (*18 s rRNA*, *GAPDH* or *β*-*actin*). Differential expression of genes was determined by the ΔΔCt method (Additional file 1).

Semiquantitative reverse-transcription PCR (RT-PCR) for the immune-suppressive genes *Indoleamine 2*,*3*-*dioxygenase* (*IDO*), *Prostaglandin E2* (*PGE2*), *hepatocyte growth factor* (*HGF*), *Transforming growth factor*-*beta* (*TGFβ*), *Galectin*, and *inducible nitric oxide synthase* (*iNOS*) was carried using 100 ng cDNA (in 5 μl volume), 2 μl of 10× standard Taq reaction buffer, 0.375 μM of forward and reverse primers, 0.2 μl 10 mM dNTPs, 1.6 μl 25 mM MgCl_2_, and 0.2 μl Taq DNA Polymerase (5 U/μl). The reaction volume was adjusted to 20 μl using water. cDNAs were amplified in a Verity 96-well thermal cycler (Applied Biosystems, USA) using the following cycling conditions: initial denaturation at 95 °C for 10 min, followed by 40 cycles of denaturation at 95 °C for 15 s, annealing and extension at 60 °C for 1 min, and a final extension at 60 °C for 1 min. The amplified PCR product was resolved on 3% agarose gel electrophoresis in TBE (tris-borate EDTA) buffer and the image was captured in the Gel Documentation system.

### Adoptive transfer of pIPCs and IPCs in NOD mice

NOD mice were obtained from The Jackson Laboratories (Barhabor, USA) and maintained in the institutional animal facility of the National Institute of Immunology. They were kept under 12 h/12 h light/dark conditions and fed *ad libitum* with autoclaved water and housed under controlled conditions of temperature and humidity. All the experiments using mice were conducted as per procedures approved by the Institutional Animal Ethical Committee (IAEC) of the National Institute of Immunology (NII), New Delhi, India.

For experimental purposes, 4-week-old NOD mice were obtained from the animal house facility, National Institute of Immunology. Blood sugar levels were measured using One Touch glucometer strips via tail vein puncture. We planned to have at least five NOD mice in each group for treatment with pIPCs, IPCs, or PBS. However, depending on the numbers of pups of the same age available at a particular time, they were divided into two groups: a control group and a treated group. Two to three independent experiments were carried out where two groups of mice were treated with pIPCs at different passages and PBS or with IPCs and PBS.

A single injection of 1 × 10^5^ pIPCs or IPCs in 50–75 μl PBS was given through the tail vein at 9 or 10 weeks of age, i.e., before the onset of clinical symptoms of T1D. For sham controls 50–75 μl PBS was injected through the tail vein. Fasting blood sugars of the mice were measured using the One Touch glucometer after 4 h of fasting every alternate week.

### Statistical analysis

The chi-squared (χ^2^) test or Fisher’s exact test was used to compare the number of mice becoming diabetic at different time points treated with either pIPCs or IPCs and controls. The Fisher’s exact test was used whenever the numbers were less than 5 in any group. In such cases odds ratios and 95% confidence intervals were calculated using Woolf’s method [24] with Haldane’s [25] modification as described previously [26]. Stata 9.2 statistical software was used to calculate χ^2^, Fisher’s exact test, odds ratios and 95% confidence intervals. A *p* value <0.05 was considered significant. Protective efficacy for pIPCs and IPCs was calculated as: (1 – odds ratio) × X 100, as described by Orenstein et al. [27]. A student’s unpaired *t* test was used to compare the delta Ct values of differentially expressed genes in pIPCs, IPCs, and control cells.

## Results

### Characterization of MSCs

#### Cell surface markers

**Fig. 1:**
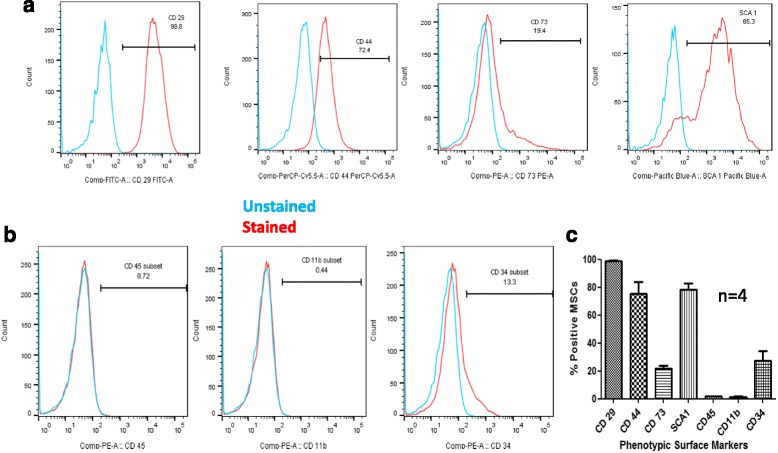
Representative FACS profile for phenotypic characterization of mesenchymal stem cells (*MSCs*). **a** Histograms showing percentage of MSCs positive for CD29 (99.8%), CD44 (72.4%), CD73 (19.4%), and SCA-1 (85.3%). **b** FACS histograms showing percentage of the hematopoietic markers CD45 (0.72%), CD11b (0.44%), and CD34 (13.3%). **c** Bar graph of four independent experiments showing percent mean ± SEM for both MSCs and hematopoietic stem cells markers: CD29 = 98.85 ± 0.33%, CD44 = 75.20 ± 8.60%, CD73 = 21.98 ± 1.81%, SCA-1 = 78.13 ± 4.64%, CD45 = 1.62 ± 0.44%, CD11b = 1.29 ± 0.54%, and CD34 = 27.40 ± 7.01% (*n* = 4)

MSCs were cultured based on their plastic adherence property as described in the Methods section. After three to four passages, homogeneous spindle-shaped MSCs were obtained (Additional file 1: Figure S1) which were characterized for their cell surface marker expression of CD29, CD73, CD44, and SCA-1; 98.85 ± 0.33% (mean ± SEM) MSCs were positive for CD29, 75.20 ± 8.60% cells were positive for CD44, 21.98 ± 1.81% cells were positive for CD73, and 78.13 ± 4.64% cells were positive for SCA-1. While the percentage of CD73-positive cells was supposed to be higher, we got an average of 21.98 ± 1.81% (Fig. 1). The hematopoietic marker CD45 was observed in 1.62 ± 0.44% of the MSCs, CD11b was observed in 1.29 ± 0.54%, and CD34 was observed in 27.40 ± 7.01% of the MSCs. The percentage of hematopoietic markers was less, as expected; however, the percentage of cells positive for CD34 was higher than expected, i.e., 27.40 ± 7.01% (Fig. 1, *n* = 4). Thus, based on the cell surface marker expression, we successfully isolated MSCs that are positive for the markers recommended by the International Society for Cellular Therapy (ISCT) [28].

### Multilineage differentiation of MSCs

**Fig. 2:**
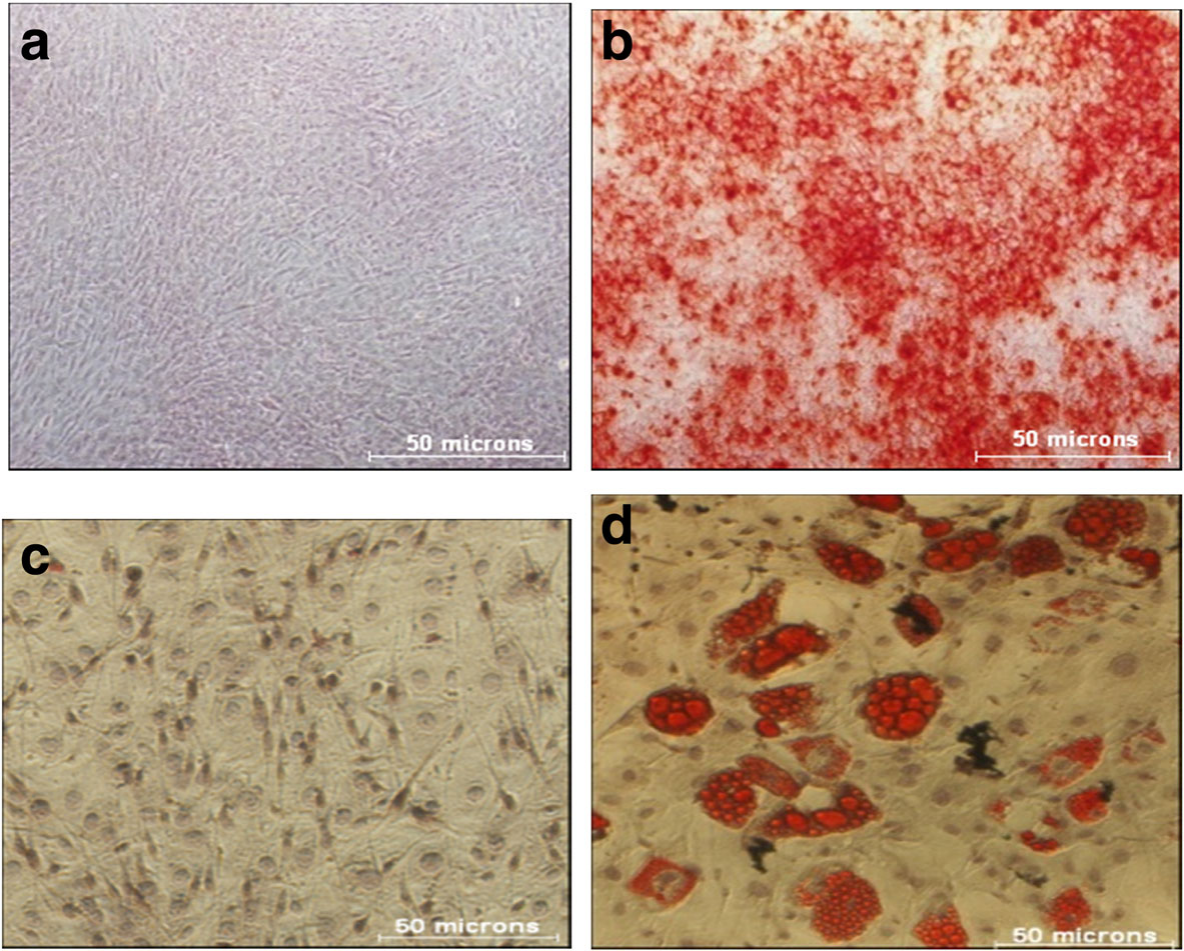
Multilineage differentiation potential of MSCs into osteocytes and adipocytes. **a** Uninduced MSCs do not stain with Alizarin *red dye*. **b** MSCs induced to become osteocytes show *red* staining with Alizarin *red dye*. **c** Uninduced control MSCs stained with Oil O *red dye* do not show any stain. **d** Differentiated adipocytes stained positive for oil droplets

Since MSCs are characterized by their multilineage differentiation potential, to further confirm their identity we differentiated them into adipocytes and osteocytes. Differentiated cells were stained with Alizarin red dye for osteocytes and with Oil red O dye for adipocytes. While the uninduced cells did not stain (Fig. 2a), differentiated osteocytes showed dark red staining with the Alizarin red dye characteristic of thick extracellular material as well as intracellular calcium deposits confirming that cells differentiated into the osteogenic lineage. Alizarin Red S stain detects the intracellular calcium, calcium-binding proteins, and proteoglycans, and thus demonstrates the mineralization in osteogenic differentiation [21, 22] (Fig. 2b). Differentiation of MSCs into adipocytes leads to the formation of oil droplets which were stained using Oil red O dye that stained the neutral triglycerides and lipids as bright red multilocular oil droplets in the induced MSCs and not in the controls (Fig. 2d and c, respectively).

### Differentiation of MSCs into IPCs

**Fig. 3:**
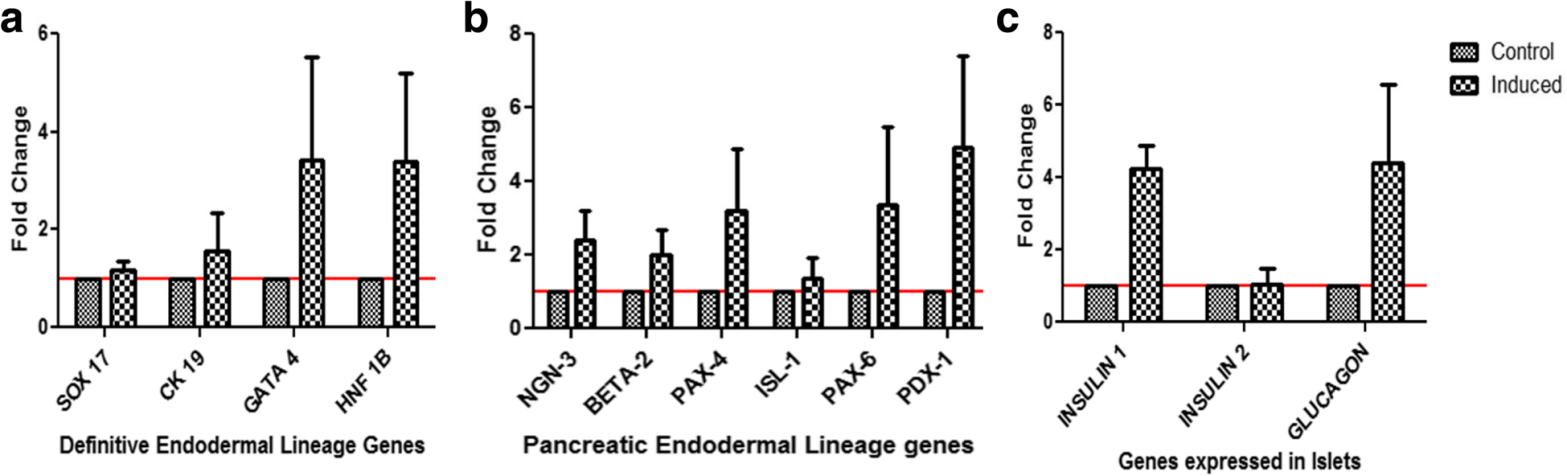
Gene expression analyses during stage-specific differentiation of insulin-producing cells using real time PCR, *n* = 3. **a** Definitive endodermal differentiation of MSCs; at day 2 of differentiation, gene expression of the transcription factors *SOX 17*, *Cytokeratin 19* (*CK 19*), *GATA 4*, and *HNF 1B* were analyzed by real-time PCR. **b** Gene expression analysis at day 5 of differentiation from the definitive endoderm to the pancreatic endoderm shows enhanced gene expression of pancreatic endodermal lineage genes *NGN*-*3*, *Beta*-*2*, *PAX*-*4*, *PAX*-*6*, and *PDX*-*1*. **c** Gene expression analysis after completion of differentiation into islet-like cells shows enhanced expression of *Insulin1* and *Glucagon* genes compared to the control

**Fig. 4:**
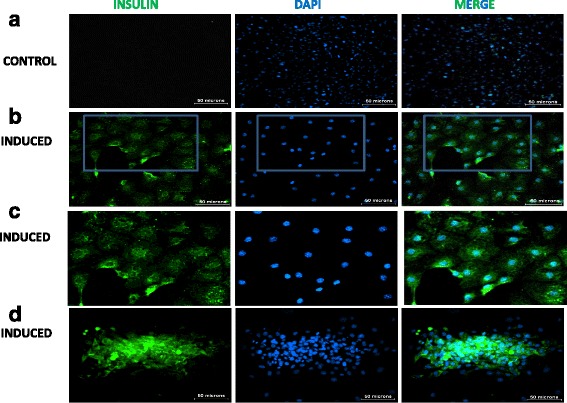
Intracellular staining for insulin. Three columns show insulin staining (*green*), DAPI staining (*blue*), and a merge of insulin and DAPI. **a** Uninduced controls showing no staining for insulin; nuclei stained blue with DAPI. **b** Induced scattered cells stained positive for insulin (*green*) **c** Magnification of the inset in (**b**) showing positive staining for insulin. **d** Differentiated islet-like clusters stained for insulin. Induced cells clearly show *green* staining for insulin in both the first column and the merged micrographs

For the differentiation of MSCs into IPCs, we adopted a new approach where we enriched the triple-positive cell population, i.e., cells expressing CD29, CD44, and SCA-1, by sorting them on FACS. For sorting, 15–40 million MSCs from passages 9 to 12 were used. MSCs were stained for CD29, CD44, and SCA-1 surface markers and sorted using FACS which resulted in enrichment of the triple-positive population from a pre-sort population of 42.0 ± 3.48% (mean ± SEM) to a post-sort population of 92.65 ± 1.76% (Additional file 1: Figure S2). These triple-positive MSCs were differentiated into IPCs using a stage-specific differentiation approach as described previously [23]. MSCs, being mesodermal in origin, were transdifferentiated towards a definitive endodermal lineage which was confirmed by the expression of definitive endodermal cell lineage genes using real-time PCR. We observed upregulation of *Cytokeratin 19* (*CK 19*), *GATA 4*, and *HNF 1B* genes while the expression of *SOX 17* gene was unaffected in the induced cultures after 2 days (Fig. 3a). These cells were further differentiated into the pancreatic endodermal lineage. Expression of transcription factors such as *Neurogenin 3* (*NGN 3*), *Beta 2*, *PAX*-*4*, *ISL*-*1*, *PAX*-*6*, and *PDX*-*1* in the differentiated cells confirmed their transdifferentiation (Fig. 3b) into the pancreatic endodermal lineage. They were further allowed to mature into islet-like cell clusters using nicotinamide, GLP-1, and taurine for 5 days in culture. After completion of the differentiation protocol, expressions of Insulin1 and Glucagon genes were found to be expressed at two or more fold higher in the induced cells compared to the uninduced control cells (Fig. 3c). Also, intracellular staining for insulin showed positive staining for islet-like clusters and scattered single cells in the induced cells while control cells, which were also grown in medium containing ITS, did not show any stain with insulin (Fig. 4). Furthermore, to confirm that insulin staining in the differentiated cells was not due to the ITS-containing medium in which they were differentiated, mouse embryonic fibroblasts (MEFs) were cultured for 10 days with or without ITS media and were stained for the presence of insulin. MEF did not stain positive for insulin under either of the conditions, suggesting that the mere presence of ITS does not result in positive staining for insulin (Additional file 1: Figure S3).

### Characterization of pIPCs

**Fig. 5:**
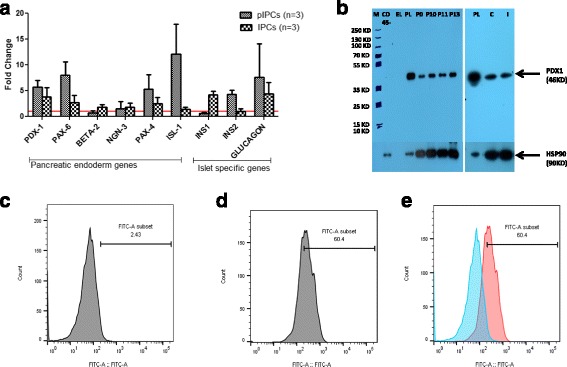
Characterization of precursors to insulin-producing cells (*pIPCs*). **a** Fold change in the expression of pancreatic lineage genes in pIPCS and insulin-producing cells (*IPCs*). The controls for calculating the fold changes in pIPCs were passage 0 cells. However, for calculating the fold change in IPCs, the controls were uninduced cells which were derived from late passages, like pIPCs (*n* = 3). **b** Western blots showing the expression of PDX1 protein at 46 kDa and loading control HSP90 at 90 kDa. Lane 1 is the molecular weight marker (*M*), lane 2 shows the cell lysate of the bone marrow-derived CD45-negative cell population as negative control (*CD*45-), lane 3 is empty (*EL*), lane 4 is pancreatic lysate (*PL*) as a positive control, followed by lysates from passage (*P*)0, 10, 11, and 13. *C* is the lysate from uninduced triple positive control cells, and *I* is the lysate from induced and differentiated IPCs. **c**–**e** Dendograms showing flow cytometric analysis for the percentage of PDX1-positive cells in pIPCs in a representative sample: **c** unstained MSCs; **d** PDX-1-FITC-stained cells; **e** a merge of PDX-1-stained and -unstained cells show that 60.4% of MSCs grown in high-glucose media were positive for PDX1. 78.65 ± 10.31% (mean ± SEM) cells were positive for PDX-1 (*n* = 3)

MSCs grown in high-glucose media for 2–3 months are expected to differentiate into pIPCs as they are known to express genes of pancreatic lineages [16]. To confirm this, we checked for the expression of the pancreatic lineage genes in cells grown in high-glucose media at passage 0, passage 11, and passage 12 using quantitative real-time PCR. We observed more than twofold higher expression of the pancreatic lineage genes *PDX1*, *PAX6*, *PAX4*, *ISL1*, *Insulin*-*2*, and *glucagon* in RNAs obtained from pIPCs compared to passage 0 cells (Fig. 5a). The controls for calculating the fold change of genes in pIPCs were passage 0 cells. However, for calculating the fold change in IPCs, the controls were uninduced cells which were derived from late passages, like pIPCs. Thus, fold change seen in IPCs is actually over and above pIPCs, although it appears to be lower in the graph when fold changes in IPCs are compared with pIPCs. Therefore, to compare the expression of the pancreatic lineage genes between pIPCs and IPCs, we plotted the scatter plot of delta Ct values (Additional file 1: Figure S4). Delta Ct values show significantly higher expression of *Beta*-*2*, *Insulin*-*1*, and *Glucagon* in IPCs compared with pIPCs; while *PDX1* expression was also higher, it was not statistically significant.

Furthermore, we checked for expression of intracellular PDX-1 (pancreatic and duodenal homeobox 1), which is a transcription factor necessary for beta cell maturation, using Western blot and Flow cytometry, confirming that these cells were indeed pIPCs. Western blot of the cells grown in high-glucose media showed a PDX-1 protein band at 46-kDa (Fig. 5b). PDX-1 protein has a molecular weight of 31 kDa, but post-translation modifications result in sumoylation of PDX-1 protein which increases the molecular weight of the protein to 46 kDa [29]. While passage 0 cells also expressed PDX1, those at the later passages had higher expression. Mouse pancreatic lysate was used as a positive control and lysate from CD45-negative bone marrow cells was used as a negative control since passage 0 cells expressed PDX1, probably due to the high-glucose medium conditions. PDX1 positivity was further confirmed by flow cytometry which revealed that 78.65% ± 10.31 (mean ± SEM) of the cells were positive for PDX1 (*n* = 3) (Fig. 5c–e). However, pIPCs were negative for intracellular staining for insulin (Additional file 1: Figure S5) despite the fact that, at the transcriptional level, they expressed the Insulin 2 gene. Thus, we were sure that the cells that were in passages 11–13 passages had become pIPCs and were not making any insulin at the protein level.

### Immunomodulatory properties of pIPCs

**Fig. 6:**
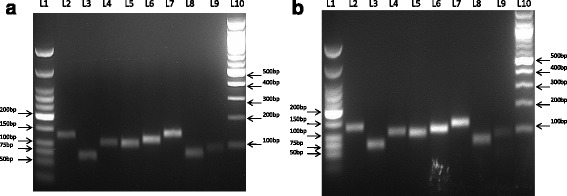
Expression of immunomodulatory genes in passage 0 cells and pIPCs using semiquantitative reverse-transcriptase PCR. **a** Expression of immunomodulatory genes in passage 0 cells. **b** Expression of immunomodulatory genes in pIPCs. Expression of *IDO*, *PGE2*, *HGF*, *TGF*-β, and *Galectin* genes was observed at both lower passage (P0) as well as in pIPCs. However, no expression of the *iNOS* gene was observed. Lane (*L*) 1: low molecular weight ladder; L2: *GAPDH* (128 bp); L3: β-*Actin* (70 bp); L4: *IDO* (109 bp); L5: *PGE2* (104 bp); L6: *HGF* (117 bp); L7: *TGF*-β (138 bp); L8: *Galectin* (77 bp); L9: *iNOS* (92 bp); L10: 100 bp ladder

MScs are well known for their immunomodulatory properties [30] which have been reported to be mediated by factors such as transforming growth factor (TGF)-β [31], nitric oxide [32], indoleamine 2,3-dioxygenase (IDO) [33], prostaglandin E2 (PGE2) [34], and so forth. Thus, we further studied the expression of various immune suppressive genes in MSCs at passage 0 and pIPCs. Semiquantitative reverse-transcriptase PCR analysis for the expression of *IDO*, *PGE2*, *hepatocyte growth factor* (*HGF*), *Transforming growth factor*-*beta* (*TGFβ*), *Galectin*, and *inducible nitric oxide synthase* (*iNOS*) revealed that all these genes except *iNOS* were expressed in MSCs right from passage 0 until passage 13 (Fig. 6), suggesting that even after passaging several times MSCs still retained their immunomodulatory properties.

### Adoptive transfer of pIPCs and IPCs in NOD mice

**Fig. 7:**
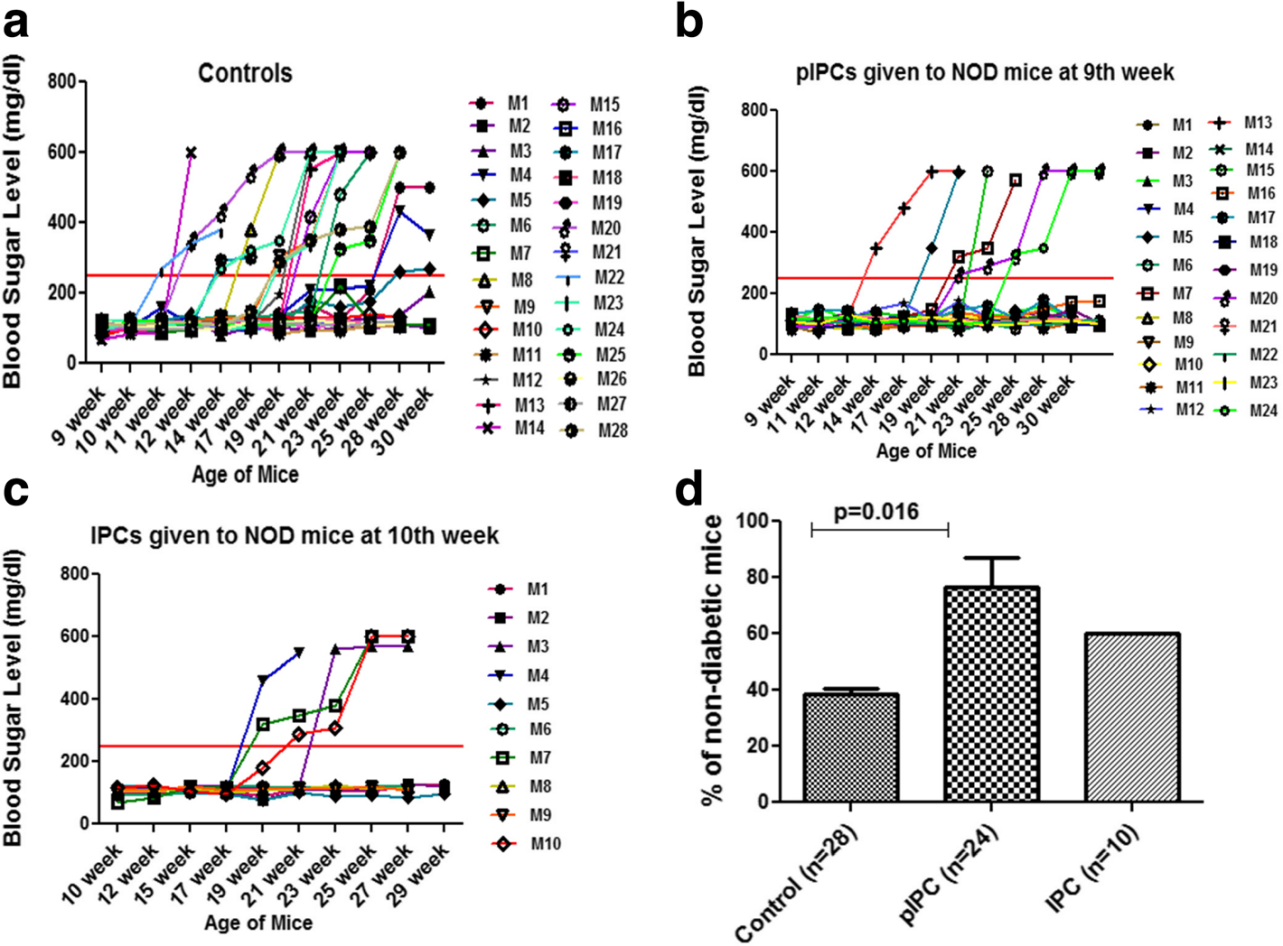
Efficacy of intravenous administration of pIPCs and IPCs at weeks 9 and 10 in preventing type 1 diabetes in nonobese diabetic (*NOD*) mice. **a** Blood glucose levels of control mice given only PBS; animals with blood glucose levels above 250 mg/dl twice consecutively were considered diabetic. The combined results of all the controls studied (*n* = 28) in the different experiments. **b** Blood glucose levels of mice given precursors to insulin-producing cells (*pIPCs*) (passage 11, 12, and 13) grown in high-glucose IMDM media (combined results of six experiments, *n* = 24). **c** Blood glucose levels of mice given differentiated insulin-producing cells (*IPCs*) at 10 weeks of age (combined result of two experiments, *n* = 10). **d** *Bar graph* showing percent of nondiabetic mice after treatment with PBS, pIPCs, and IPCs: 38.75 ± 1.99% (mean ± SEM) of NOD mice (*n* = 28) in the control group remained nondiabetic, 76.67 ± 10.54% NOD mice (*n* = 24) receiving pIPCs remained nondiabetic, and 60.0 ± 0% of NOD mice (*n* = 10) receiving differentiated IPCs remained nondiabetic

NOD mice were treated with pIPCs from passages 11, 12, and 13 and differentiated IPCs at 9 weeks of age (i.e., before the expected time of manifestations of diabetic symptoms), and were followed-up for fasting blood glucose levels till weeks 29–30 using the One-Touch glucometer. Mice with fasting blood sugar levels of more than 250 mg/dl twice consecutively were considered diabetic. As is clear from Fig. 6, only 38.75 ± 1.99% of the control mice were nondiabetic at the end of weeks 29–30 (Fig. 7a and d). However, a significantly higher percentage (76.67 ± 10.54%) of mice (*p* = 0.016) treated with pIPCs (combined data for passage 11, 12, and 13) remained nondiabetic (Fig. 7b and d). Of the mice treated with differentiated IPCs (Fig. 7c and d), 60 ± 0% were nondiabetic at the end of weeks 29–30, although the difference was not statistically significant when compared to control mice.

### Protective efficacy of pIPCs and IPCs

To study the protective efficacy, we combined all three groups of NOD mice who received pIPCs, i.e., passage 11, 12, and 13 MSCs. Similarly mice from two experiments that received IPCs were also combined, and all control mice from all the experiments were combined. The number of mice becoming diabetic in the group that received pIPCs or IPCs were compared with the controls, and the percent protective efficacy was calculated from the odds ratios: (1 – odds ratio) × 100 [27]. A significant difference started appearing between the mice treated with pIPCs and controls as early as week 14, when 17.86% of the control mice became diabetic compared to none in pIPC and IPC groups. Both IPCs and pIPCs delayed the onset of diabetes (Table 1). We observed a protective efficacy of 78.4% as early as week 28 when pIPCs were given to the mice as compared to a protective efficacy of only 55% when IPCs were given (Table 1). At weeks 29–30, while about 61% of control NOD mice became diabetic, 25% of those who received pIPCs and 40% of those who received IPCs became diabetic, suggesting a benefit of pIPCs over IPCs.

**Table 1:** Protective efficacy of pIPC and IPC treatment in NOD mice followed-up for 29–30 weeks

Weeks	Number of diabetic mice in the pIPC group (*n* = 24)	Number of diabetic mice in the IPC group (*n* = 10)	Number of diabetic mice in the control group (*n* = 28)	pIPC vs control *p* OR (95% CI)	Protective efficacy of pIPCs (1 – OR) × 100	IPC vs control *p* OR (95% CI)	Protective efficacy of IPCs (1 – OR) × 100
9 weeks	0 (0%)	0 (0%)	0 (0%)	–	–	–	–
10 weeks	0 (0%)	0 (0%)	0 (0%)	–	–	–	–
12 weeks	0 (0%)	0 (0%)	3 (10.7%)	*p* < 0.14 OR = 0.14 (0.2–1.25)	86%	*p* < 0.39 OR = 0.35 (0.04–2.99)	65%
14 weeks	0 (0%)	0 (0%)	5 (17.86%)*	*p* < 0.03 OR = 0.087 (0.01–0.7)	91.3%	*p* < 0.19 OR = 0.2 (0.02–1.68)	80%
16 weeks	1 (4.2%)	0 (0%)	6 (21.43%)	*p* < 0.07 OR = 0.22 (0.06–0.83)	78%	*p* < 0.13 OR = 0.16 (0.02–1.34)	84%
18 weeks	3 (12.5%)*	2 (20%)	9 (32.14%)	*p* < 0.08 OR = 0.33 (0.13–0.88)	67%	*p* < 0.39 OR = 0.6 (0.19–1.88)	40%
20 weeks	4 (16.2%)	3 (30%)*	12 (42.86%)	*p* < 0.039 OR = 0.29 (0.12–0.7)	71%	*p* < 0.37 OR =0.62 (0.22–1.73)	38%
22 weeks	5 (20.8%)	4 (40%)	14 (50%)	*p* < 0.02 OR = 0.26 (0.06–1.03)	74%	*p* < 0.43 OR =0.69 (0.26–1.87)	31%
24 weeks	6 (25%)	4 (40%)	14 (50%)	*p* < 0.06 OR = 0.33 (0.08–1.24)	67%	*p* < 0.43 OR =0.69 (0.26–1.87)	31%
26 weeks	6 (25%)	4 (40%)	14 (50%)	*p* < 0.06 OR = 0.33 (0.08–1.24)	67%	*p* < 0.43 OR =0.69 (0.26–1.87)	31%
28 weeks	6 (25%)	4 (40%)	17 (60.71%)	*p* < 0.009 OR = 0.216 (0.05–0.82)	78.4%	*p* < 0.22 OR = 0.45 (0.17–1.24)	55%
29/30 weeks	6 (25%)	4 (40%)	17 (60.71%)	*p* < 0.009 OR = 0.216 (0.05–0.82)	78.4%	*p* < 0.22 OR = 0.45 (0.17–1.24)	55%

Values are shown as *n* (%)

*Cumulative numbers of mice becoming diabetic are shown over time

*CI* confidence interval, *IPC* insulin-producing cell, *OR* odds ratio, *pIPC* precursor to insulin-producing cell

## Discussion

In the present study, MSCs were cultured from Balb/c mouse bone marrow and characterized based on ISCT guidelines [28]. MSCs are defined by their fibroblastic spindle-shaped morphology and thus are morphologically indistinguishable from fibroblasts. Also they do not have a single defined phenotypic marker that would unambiguously characterize them and differentiate them from fibroblasts or any other contaminating cells in the culture. Therefore, according to ISCT guidelines, one of the criteria to be met by MSCs is the presence of a defined set of phenotypic cell surface markers such as CD29, CD74, CD44, and SCA-1, and ≤2% expression of hematopoietic cell surface markers CD45, CD34, and CD11b. These guidelines are defined for human MSCs; however, for nonhuman MSCs a plastic adherence property as well as multilineage differentiation potential remains applicable, but expression of surface markers may vary with MSCs from different sources [28]. While the frequencies of CD29-, CD44-, and SCA-1-positive cells were similar to the expected frequencies, only 21.98 ± 1.81 of MSCs were positive for the CD73 cell surface marker. CD73 expression is considered to be an important surface marker for defining human MSCs [28]; however, for murine MSCs there are controversial reports showing both the presence in small number of MSCs as well as the absence of CD73 on murine MSCs [23, 35].

As expected, less than 2% of MSCs in our cultures were expressing hematopoietic markers CD45 and CD11b. CD34 expression, however, was found in 27.4% of MSCs. MSCs were initially believed to be negative for CD34, but recently there are reports showing that MSCs originate from CD34^+^ progenitor stem cells and these cells lose expression of CD34 due to in-vitro culture conditions suggesting that MSCs may not be truly negative for CD34 expression [36–38]. Simmons and Torok-Storb [36] have convincingly shown that bone marrow-derived MSCs are CD34^+^ since 95% of the detectable fibroblastic colonies originated from bone marrow cells sorted based on CD34 expression. Furthermore, Kaiser et al. [37] demonstrated the ability of a CD34^+^ fraction of cells to differentiate into osteocytes, adipocytes, and chondrocytes. These studies suggest that MSCs originated from CD34^+^ cells and thus CD34 cannot be considered as a true negative marker for MSCs. CD34 expression has also been correlated with the higher vasculogenic and angiogenic potential of MSCs in vivo [39]. They differentiate into endothelial cells in vivo and help in neovascularisation which, in turn, helps in the establishment of the regenerative microenvironment.

Several studies have shown differentiation of MSCs into insulin-producing cells and their use in ameliorating diabetes in streptozotocin-induced diabetes in rats or mice [19, 40, 41]. However, there are several unanswered questions, such as what is the lifespan of the differentiated cells, whether they would further multiply or die after some time following in-vivo infusions, whether one needs to give a booster of differentiated cells after the initial intravenous administration, and finally whether these cells can be used to prevent diabetes in animals or humans predisposed to become diabetic. Several studies have been conducted in type 1 diabetes patients where MSCs derived from adipose tissue [42], Wharton’s jelly [43], or bone marrow [44], either differentiated into insulin-producing cells or undifferentiated, were given to the patients. However, these studies showed variable results with limited outcomes. When we tried to grow the differentiated insulin-producing cells in culture, they did not survive for a long time (unpublished observations) suggesting that, once differentiated, they may not divide any further and may have a limited lifespan. This observation raised the question whether MScs should be differentiated and then given to NOD mice or whether the precursors to beta cells should be given. To resolve this issue, we have grown MSCs in high-glucose medium for 11, 12, and 13 passages, and confirmed the expression of PDX-1 (pancreatic and duodenal homeobox 1), which is a transcription factor necessary for beta cell maturation, using Western blot and FACS analysis to assure ourselves that those cells were indeed precursors to insulin-producing cells (pIPCs). Besides PDX1, pIPCs also expressed other pancreatic lineage genes such as *PAX6*, *PAX4*, *ISL1*, *Insulin*-*2*, and *Glucagon* at the transcriptional level, but were not positive for insulin staining. These pIPCs were then given to NOD mice before the onset of clinical manifestations of T1D and compared with the group of mice that received MSCs differentiated into insulin-producing cells and controls to compare the efficacy of the two modalities to arrest diabetes in NOD mice. Our results show a better response when pIPCs were given to NOD mice: 76.67 ± 10.54% of NOD mice remained nondiabetic when treated with passage 11, 12, and 13 MSCs compared to when differentiated insulin-producing cells were given (60% in two experiments). While differentiated insulin-producing cells did give better results than controls (where only 38.75% of the mice were nondiabetic at the end of weeks 29–30), precursors to insulin-producing cells definitively gave much better results compared with control (*p* < 0.016). Ezquer et al. reported that a single dose of undifferentiated bone marrow-derived MSCs could revert hyperglycemia and glycosuria to normal levels in mice induced with type 1 diabetes using streptozotocin [19]. However, while they evaluated the therapeutic potential of MSCs, we have evaluated the immunoprophylactic efficacy of MSCs grown in high-glucose media for a long time which could be called precursors to insulin-producing cells. While 60–80% of female NOD mice have been reported to spontaneously develop human type 1 diabetes-like symptoms by the age of 20 weeks [17], in our study 42.86% of the control mice became diabetic by 20 weeks and 60.71% became diabetic at weeks 29–30. This could be due to the environmental conditions maintained in our small animal facility (Table 1).

Several induction protocols have been reported in the literature for the differentiation of MSCs into insulin-producing cells [45–50]. We tried some of these protocols but did not achieve a very high percentage of insulin-producing cells (unpublished observations). Chandra et al. [23] reported very good results from adipose tissue-derived single-cell clones of MSCs that were positive for CD29, CD44, and SCA1. However, instead of cloning the cells, we decided to use a novel strategy—to sort the bone marrow-derived MSCs cultured for 10–13 passages, based on these cell surface markers (CD29, CD44, and SCA1), which saved the time that would have gone into cloning of the cells. The triple-positive cells were then differentiated into insulin-producing cells using the stage-specific differentiation protocol described by Chandra et al. [23] with a few modifications. MSCs were differentiated in tissue culture plates instead of low adherent plates, and culture as well as differentiation was carried out in IMDM instead of Dulbecco’s modified Eagle’s medium (DMEM). The cells were first differentiated to form definitive endoderm followed by pancreatic endoderm and, finally, islet-like cell clusters. However, despite getting a very high percentage of cells staining for insulin and islet-like clusters, the in-vivo infusions did not show very promising results as 40% of the mice still became diabetic, while 76.67 ± 10.54% of the NOD mice treated with precursors to insulin-producing cells were nondiabetic at the end of weeks 29–30. It seems that, after differentiation, insulin-producing cells do not have a very long lifespan in vivo, and thus we may need to give more than one infusion of these cells to completely ameliorate diabetes in NOD mice. However, precursors which have been grown under a high-glucose concentration seem to be more protective. It is also possible that once differentiated into insulin-producing cells, MSCs lose their immunomodulatory properties, are not able to regulate the autoimmune response, and succumb to autoimmune attack by T cells. However, precursors still maintain their immune-regulatory properties as evident from the expression of immunomodulatory genes and may thus be able to attenuate the autoimmune responses, resulting in more diabetes-free animals in such groups. These results may further be improved if at least two infusions of precursors are given before the onset of clinical symptoms. MSCs grown in high-glucose medium for a long duration start expressing the transcription factors that are expressed in the precursors of beta cells, and with different chemical treatments in vitro these cells can be matured towards beta-like cells [16]. However, when injected in NOD mice, these precursor cells may be reaching the site of damage, i.e. the pancreas, downregulating the autoimmune responses, and probably differentiating in vivo into insulin-producing cells due to the microenvironment of the pancreas and may have better survival and are thus more protective. However, intravenously infused MSCs have been reported to initially home to the lungs and are known to move within 24 h [51] to either the injured site or to organs such as the liver, kidney, and spleen [52–55]. When they reach the site of injury, they might pass on their effects to other cells and thus carry out their therapeutic effect. It has been observed that these cells induce regulatory T cells and regulatory macrophages, which help in carrying out the immunomodulatory and regenerative effects of MSCs [56]. More studies using multiple infusions of the precursors to insulin-producing cells at different ages of NOD mice will help design protocols for clinical use of these cells in high-risk groups for type 1 diabetes.

## Conclusions

Our results suggest that MSCs grown in high-glucose media for 11–13 passages exhibit the characteristics of precursors to insulin-producing cells and, when administered to NOD mice before the clinical onset of symptoms, help in preventing type 1 diabetes-like symptoms in the mice. Insulitis and beta cell damage start as early as 4–7 weeks, and the clinical manifestations are observed only when more than 90% of the cells are destroyed. However, when the pIPCs are given at week 9, they probably home to the site of the damage, prevent further autoimmune attack by infiltered autoimmune cells, and differentiate into insulin-producing cells due to the pancreatic microenvironment. These results may further be improved if the first administration of pIPCs is performed at weeks 4–5 week followed by another one at week 9 week. When translated to humans, autologous MSCs grown in high-glucose media for 10–13 passages may have beneficial effects in individual at high risk of developing type 1 diabetes.

## Additional file

Additional file 1: Contains supplementary methods and supplementary figures. (PDF 803 kb)

## Abbreviations

BSA: Bovine serum albumin
ESC: Embryonic stem cell
FACS: Fluorescence-activated cell sorting
FBS: Fetal bovine serum
FITC: Fluorescein isothiocyanate
GLP: Glucagon-like peptide
IBMX: Isobutyl-1-methylxanthine
IMDM: Iscove’s modified Dulbecco’s medium
IPC: Insulin-producing cell
ISCT: International Society for Cellular Therapy
ITS: Insulin-transferrin-selenium
MEF: Mouse embryonic fibroblast
MSC: Mesenchymal stem cell
NEAA: Nonessential amino acid
NFDM: Nonfat dry milk
NOD: Nonobese diabetic
PBS: Phosphate-buffered saline
PCR: Polymerase chain reaction
PE: Phycoerythrin
pIPC: Precursor to insulin-producing cell
SFM: Serum-free medium
T1D: Type 1 diabetes
