# Supplementary material for: Do we really need to differentiate mesenchymal stem cells into insulin-producing cells for attenuation of the autoimmune responses in type 1 diabetes: immunoprophylactic effects of precursors to insulin-producing cells
Source: Stem Cell Res Ther. 2017 Jul 12;8:167. doi: 10.1186/s13287-017-0615-1 (PMC5508489; doi:10.1186/s13287-017-0615-1)
Supplement: Additional file 1: — Contains supplementary methods and supplementary figures. (PDF 803 kb) [file 13287_2017_615_MOESM1_ESM.pdf]

Do we really need to differentiate Mesenchymal Stem Cells into Insulin producing cells for attenuation of autoimmune responses in Type 1 Diabetes: Immunoprohpylactic effects of precursors to insulin producing cells?

Anshu Sharma[1] & Rajni Rani[1,2@]

1. Molecular Immunogenetics Group
   National Institute of Immunology
   New Delhi-110067
   India

2. Systems Biology Group,
   CSIR- Institute of Genomics and Integrative Biology,
   New Delhi-110025,
   India.

@ Correspondence may be addressed to

Dr. Rajni Rani
Systems Biology Group,
Institute of Genomics and Integrative Biology (CSIR),
New Delhi-110025, India.
Email: rajni@nii.ac.in, rajni.rani@igib.in

Supplementary Materials

Supplementary Materials and methods

Immunostaining of Mouse Embryonic Fibroblasts (MEFs) for insulin

To ascertain that the insulin producing cells were not staining just because they were grown in medium containing insulin, we cultured mouse embryonic fibroblasts [24] for 10 days in the presence as well as absence of ITS (Insulin transferrin and Selenium) in the culture media (DMEM-HG with 10% FBS). After ten days cells were stained for the presence of intracellular insulin. Briefly, the cells were washed using PBS and fixed in 4% paraformaldehyde for 10 min at room temperature. After fixation, cells were washed with PBS followed by Permeabilzation using 0.25% Triton X-100 in PBS for 10 min and blocked using 1% BSA in PBS for 1 hour at room temperature. For the presence of intracellular insulin, cells were incubated overnight with anti insulin rabbit polyclonal antibody at 1:200 dilution (Santa Cruz SC-9168), washed with PBS and stained with secondary antibody Alexa Fluor 488 goat anti rabbit (Invitrogen) for 1 hour at room temperature at 1:500 dilution. After another PBS wash cells were counter stained for nucleus with DAPI (molecular probes) and observed under fluorescent microscope

Sorting of CD45-ve cell population from the bone marrow

Bone marrow cells were isolated from Balb/c mouse bone marrow as described in Materials and Methods section. The cells thus obtained were resuspended in plane IMDM media and sorted using Fluorescence Activated Cell Sorter (FACS) to obtain cells negative for CD45 phenotypic marker (CD45-ve cell population). To achieve this, bone marrow cells were stained with Fluorescein isothiocyanate conjugated anti-mouse CD45.2 (eBiosciences) for 30 minutes at 4°C. After staining, cells were passed through 70µm cell strainer and centrifuged at 300g for 5mins. Cells were resuspended in plane IMDM and were sorted for CD45-ve cell population in Fluorescence Activated Cell Sorter (FACS) (Aria cell sorter).

Isolation and purification of RNA from Tri-reagent

RNA was isolated from the MSCs using Tri-reagent following manufacturer's protocol. Briefly, cells were homogenized in Tri-reagent and 1/5th the volume of chloroform was added and mixed thoroughly till milky white precipitation was formed. Then samples were allowed to stand for 10 minutes for separation of aqueous and organic layers formation and centrifuged at maximum RPM for 15 minutes at 4 °C. The aqueous supernatant was collected in a fresh tube, and precipitated with isopropanol (½ the volume of original Tri-reagent) in -20 freezer for two hours. The RNA was pelleted by centrifugation at maximum RPM and pellet was washed with 70% ethanol and air-dried. For DNase I treatment and column purification we have used Qiagen RNeasy Mini Kit using manufacturer's instructions. Briefly, the pellet was re-suspended in 88.5 µl RNase-free water and 10 µl of RDD buffer and 2.5 µl of Rnase-free Dnase I was added and incubated at RT for 15 minutes. To this DNase I treated RNA, 350 µl RLT buffer and 250 µl absolute ethanol were added, mixed and immediately transferred onto the RNeasy columns. Flow-through was removed by centrifugation and the column was washed with 500 µl RPE wash buffer twice and all traces of the RPE were removed with an empty spin. For elution of RNA, 40 µl of RNase-free water which was preheated at 55 °C was added to the column and incubated for 10 minutes, and spun-down into fresh nuclease free vials. RNA was quantitated using absorbance at 260 nm using the Nanodrop and run on a 1% agarose gel in TBE to determine RNA integrity checks.

cDNA synthesis

RNA was reverse transcribed using Superscript III cDNA synthesis kit (Invitrogen) according to manufacturer's protocol. Briefly, to 500 ng – 4000 ng of RNA (in 8µl volume), 1 µl (50 ng) random-hexamer mix or 1 µl (50 µM) oligo dT and 1 µl (1mM) dNTP mix were added and incubated at 65°C for 5 min and snap-chilled on ice for at least one minute. The cDNA synthesis

was initiated with 200 units of Superscript III enzyme (1 µl) with 5mM $MgCl_2$ (4 µl of 25mM $MgCl_2$), 1x RT buffer (2 µl), 1mM DTT (2 µl of 0.1M), and 40 units of RNaseOUT (1 µl) in a total volume of 20µl. Annealing was carried out at 25°C for 10 min followed by an extension at 50°C for 1 h. RNA was hydrolyzed using RNase H (1 µl) for 20 min at 37°C.

Real Time PCR data analysis

Differential expression was determined by the ΔΔCt method. Mean Ct values were calculated for the triplicates. Replicates that deviated by more than 0.5 Ct were excluded from the analysis. Mean Ct values obtained for target genes were normalised to the corresponding 18s rRNA, GAPDH or β-actin. ΔCt was calculated by subtracting the 18s rRNA, GAPDH or β-actin Ct value from the target gene Ct. Fold change was determined by using the formula $2^{-\Delta\Delta Ct}$ where ΔΔCt was Induced ΔCt – Control ΔCt. The reference sample would have a fold change of 1.

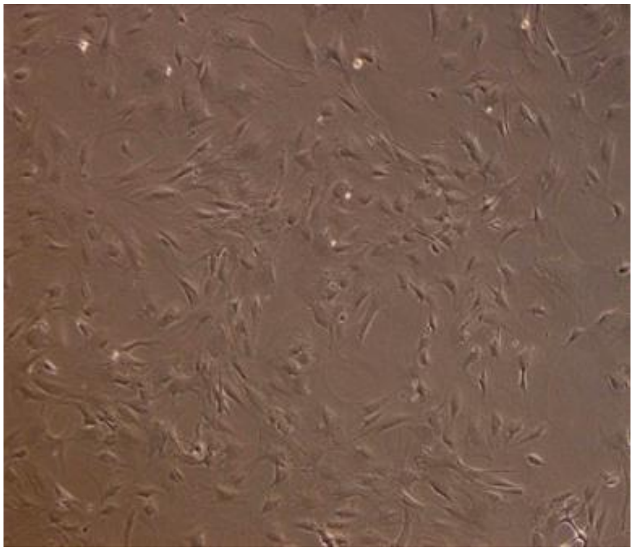

Supplementary Figure 1: Morphology of enriched homogeneous spindle shaped Mesenchymal stem cells cultured from mouse bone marrow obtained after three to four passages in culture.

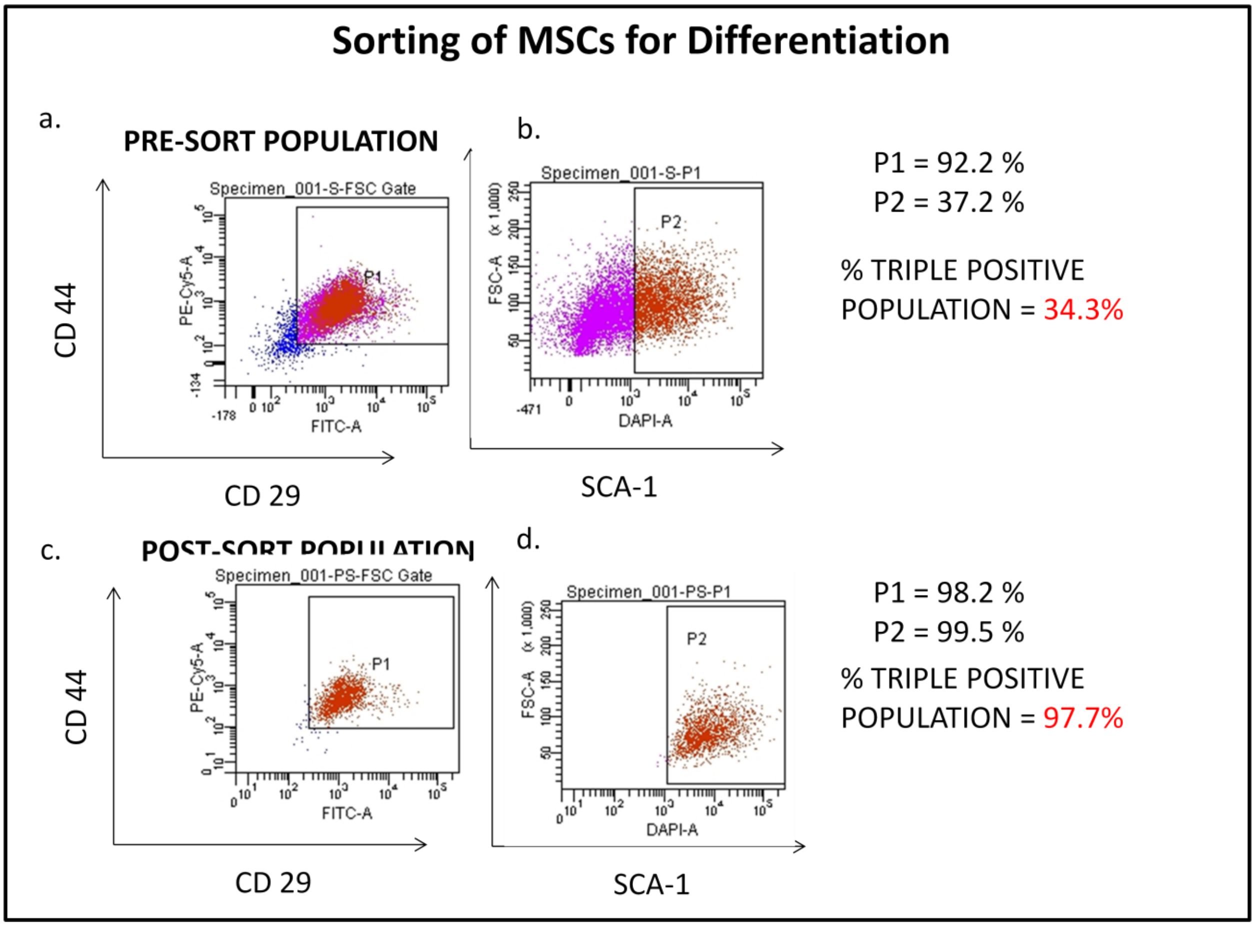

Supplementary Figure 2. Sorting of MSCs for triple positive cell surface markers: CD29, CD44, SCA-1. **a.** FACS plot for CD29 and CD44 double positive cells. 92.2% of the cells in this representative figure are positive for both the markers. (Gate P1) **b.** These double positive cells were seen for Sca-1 marker and 37.2% of the 92.2% double positive cells were found to be positive for Sca-1, thus the percentage of total triple positive cells was 34.3% cells. (Gate P2, selected within P1 Gate) **c**. Evaluation of percent positivity for CD29 and CD44 in post sort population, 98.2% of the cells were double positive (Gate P1) and **d.** 99.5% of the double positive cells were also positive for Sca-1, thus the percentage of total triple positive cells post sorting was 97.7% (Gate P2, selected within P1 Gate)

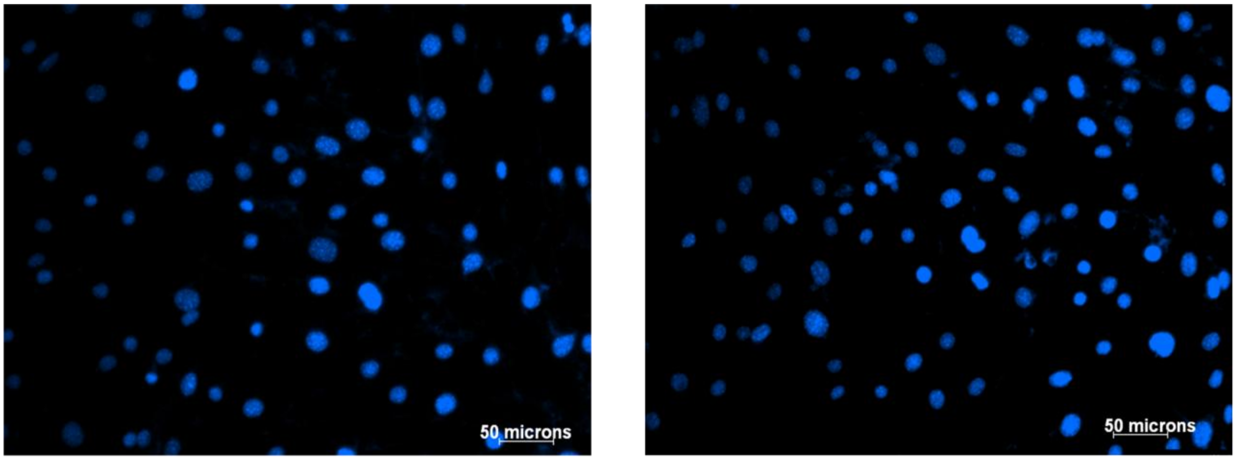

a.) MEF WT3T3 cells cultured without ITS b.) MRF WT3T3 cells cultured with ITS

Supplementary Figure 3: Mouse embryonic fibroblast (MEF) cell line WT3T3 cultured in the presence as well as absence of ITS and stained for the presence of insulin. a. cells cultured without ITS. b) cells cultured in the presence of ITS. No positive staining for insulin was observed in the cells grown with or without ITS. Nuclei are stained blue with DAPI.

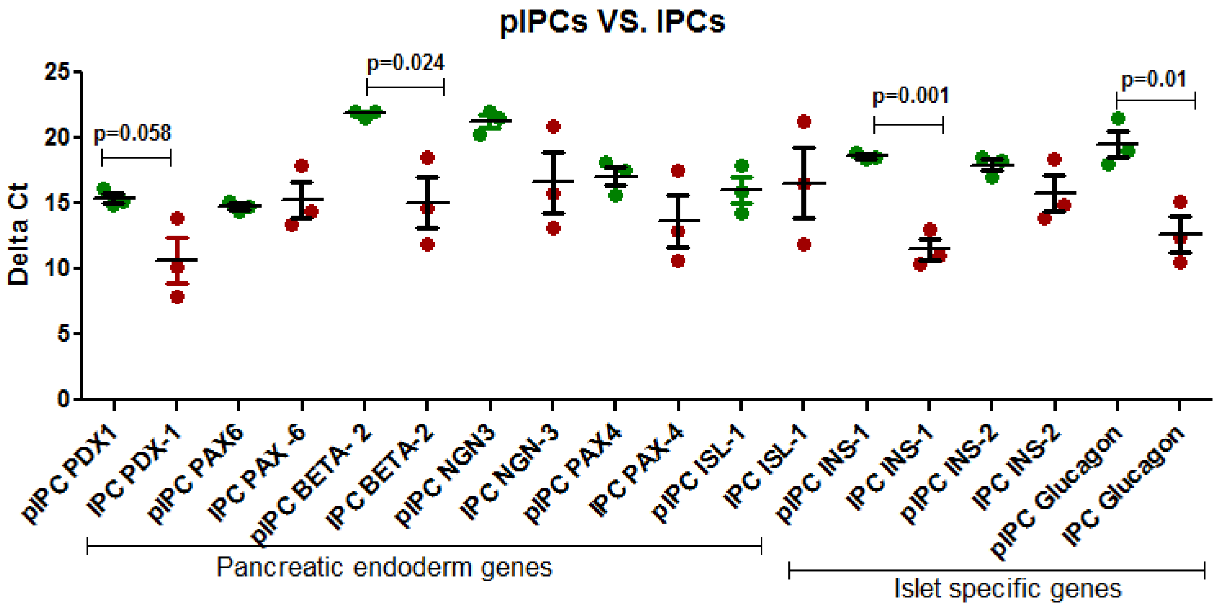

Supplementary Figure 4. Comparison of the delta Ct values obtained for pancreatic endoderm and islet specific genes in pIPCs and IPCs. Lower Delta Ct values for IPCs shows higher expression of the genes compared to pIPCs. Statistically Significantl higher expression of Beta-2, Insulin-1 and Glucagon was observed in IPCs compared with pIPCs. While PDX1 expression is higher in IPCs compared with pIPCs, the difference is not statistically significant.

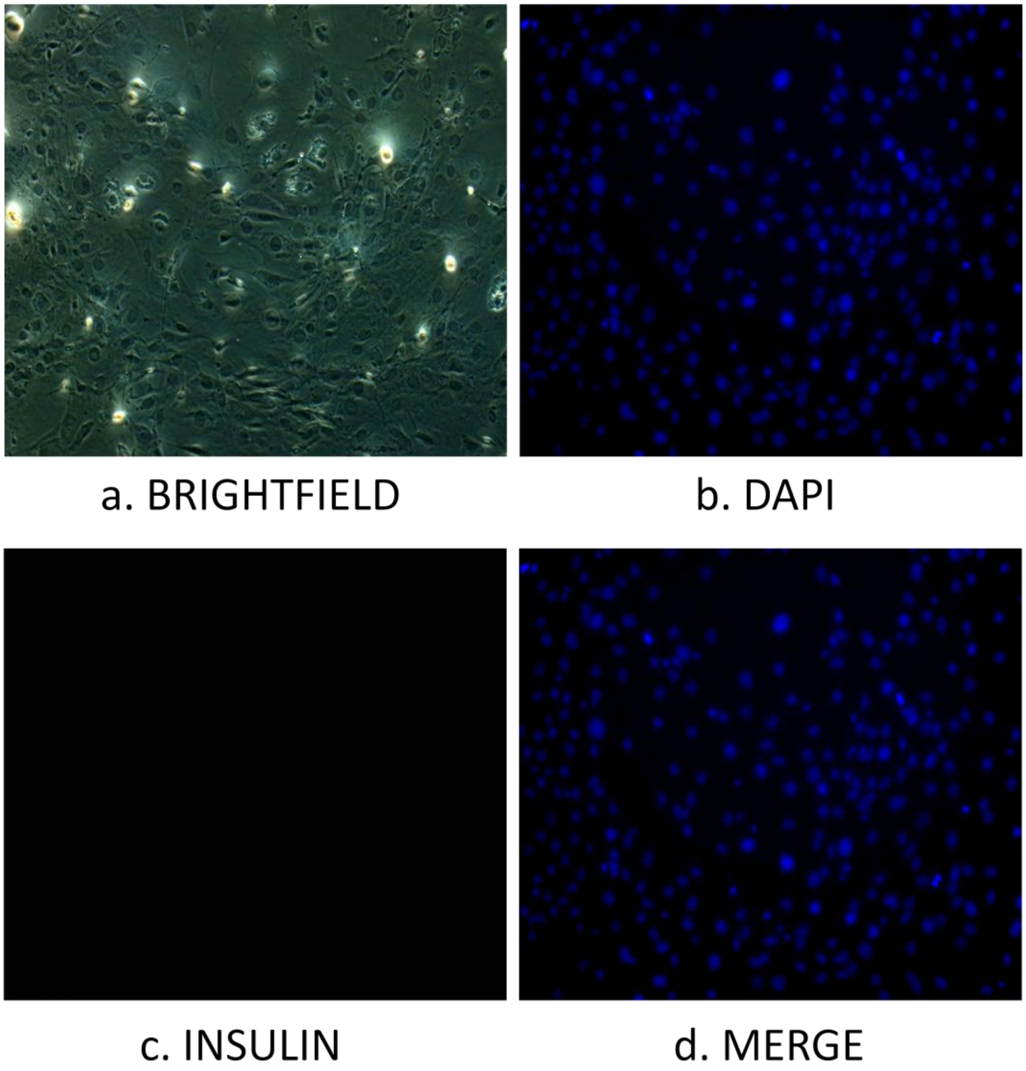

Supplementary Figure 5. Intracellular staining for insulin in pIPCs. **a.** Brightfield image of the cells. **b.** Nuclei stained positive with DAPI. **c.** pIPCs stained negatively for insulin. **d.** Merge image showing the staining for DAPI and none of the cells are stained positive for insulin.
